# Characterization of transcriptional response of *Lactobacillus plantarum* under acidic conditions provides insight into bacterial adaptation in fermentative environments

**DOI:** 10.1038/s41598-020-76171-6

**Published:** 2020-11-05

**Authors:** Sera Jung, Jong-Hee Lee

**Affiliations:** Advanced Process Technology and Fermentation Research Group, Research and Development Division, World Institute of Kimchi, Gwangju, 61755 Republic of Korea

**Keywords:** Microbiology, Molecular biology

## Abstract

Lactic acid bacteria (LAB) play an important role in kimchi fermentation by metabolizing raw materials into diverse metabolites. Bacterial adaptation is therefore a crucial element of fermentation. In this study, we investigated the transcriptional changes of *Lactobacillus plantarum* under acidic conditions to evaluate the elements of bacterial adaptation critical for fermentation. Differentially expressed genes (DEGs) have shown that transport function is primarily affected by acidic conditions. Five of the 13 significantly down-regulated genes and 7 of the 25 significantly up-regulated genes were found to have transport-related functions. We quantified the intracellular leucine content of bacteria grown at different pH ranges, determining that optimal bacterial leucine transport could be controlled by acidity during fermentation. Inhibition of *L. plantarum* growth was investigated and compared with other LAB at a pH range of 6.2–5.0. Interestingly, valinomycin inhibited *L. plantarum* growth from pH 6.2 to 5.0. This showed that *L. plantarum* had a wider range of transport functions than other LAB. These results suggested that *L. plantarum* had robust transport functions, and that this was the crucial factor for bacterial adaptation during fermentation.

## Introduction

Kimchi is a well-known fermented food mainly comprising salted cabbage, radish, ginger, garlic, and red pepper powder. Kimchi and fermented foods have been reported to have beneficial effects on health, and this is believed to be associated with probiotic lactic acid bacteria (LAB) and postbiotic fermented metabolites^1–4^.

A sour taste is one of the most characteristic features of kimchi, and is mainly caused by the production of organic acid during fermentation. The pH at the initial stage is around 6 and decreases to pH 4 at the fourth week of fermentation^5^. Lactic acid is most abundant organic acid involved in kimchi fermentation, and is produced by hetero- and homo-fermentative LAB^1^. The lactic acid content and composition could be affected by the pasteurization temperature and storage conditions^6,7^. The lactic acid content was approximately 50–70% of the total organic acid, and had a higher concentration when kimchi was stored at 10 °C than at 4 *°*C^7^.

Acidification is an important aspect of kimchi fermentation, as it prevents the growth of undesired bacteria. However, it also affects the dynamics of LAB during kimchi fermentation, due to the difference in acid tolerance among the LAB groups.

The genera *Leuconostoc*, *Weissella*, and *Lactobacillus* are the major groups of LAB involved in kimchi fermentation^1,5,8,9^. LAB contribute to the conversion of raw materials into the metabolites which confer the taste of fermented foods^10,11^. The process of kimchi fermentation and the mechanisms of lactic acid bacterial adaptation involved therefore need to be further characterized.

The genus *Lactobacillus* is the dominant group involved in kimchi fermentation, producing the lactic acid. *L. plantarum* is beneficial to both food fermentation and human health, and has been designated as a probiotic bacterium by the Korea Ministry of Food and Drug Safety (MFDS). *L. plantarum* has been reported to have diverse functionality. Antibacterial activity from *L. plantarum* was demonstrated to contribute to food preservation and safety^12–14^, and has been found to have probiotic features^15,16^. Further, *L. plantarum* prevents intestinal inflammatory disease by controlling cytokine expression^17^. It has also been isolated from a variety of sources, indicating versatile adaptation in diverse environments^18^. Bacterial transcriptional analysis provides insight on bacterial gene expression in diverse environmental conditions^18,19^.

Studies on *L. plantarum* transcription upon exposure to hydrogen peroxide and *p*-coumaric acid revealed up- and down-regulation of detoxification function and metabolic functions^18,20,21^. Gallic acid treatment increased the expression of transport-related genes and was correlated with the proton motive force (PMF) across the membrane^22^. Transcriptional analysis of *L. plantarum* adapted at different model such as pineapple and carrot juice, showed that 21–31% of the genes were differently expressed, depending on the plant niche and physiological state of the cell. In both the model systems, *L. plantarum* showed a large number of up-regulated genes that correspond to carbon and nitrogen metabolism^18^.

The gene expression of LAB under acidic conditions must therefore be characterized to evaluate bacterial adaptation and subsequent metabolite production. In this study, we analyzed *L. plantarum* gene expression under acidic conditions to identify and understand the important elements of their adaptation in fermentative environments.

## Results

### *L. plantarum* transcriptome analysis

**Figure 1:**
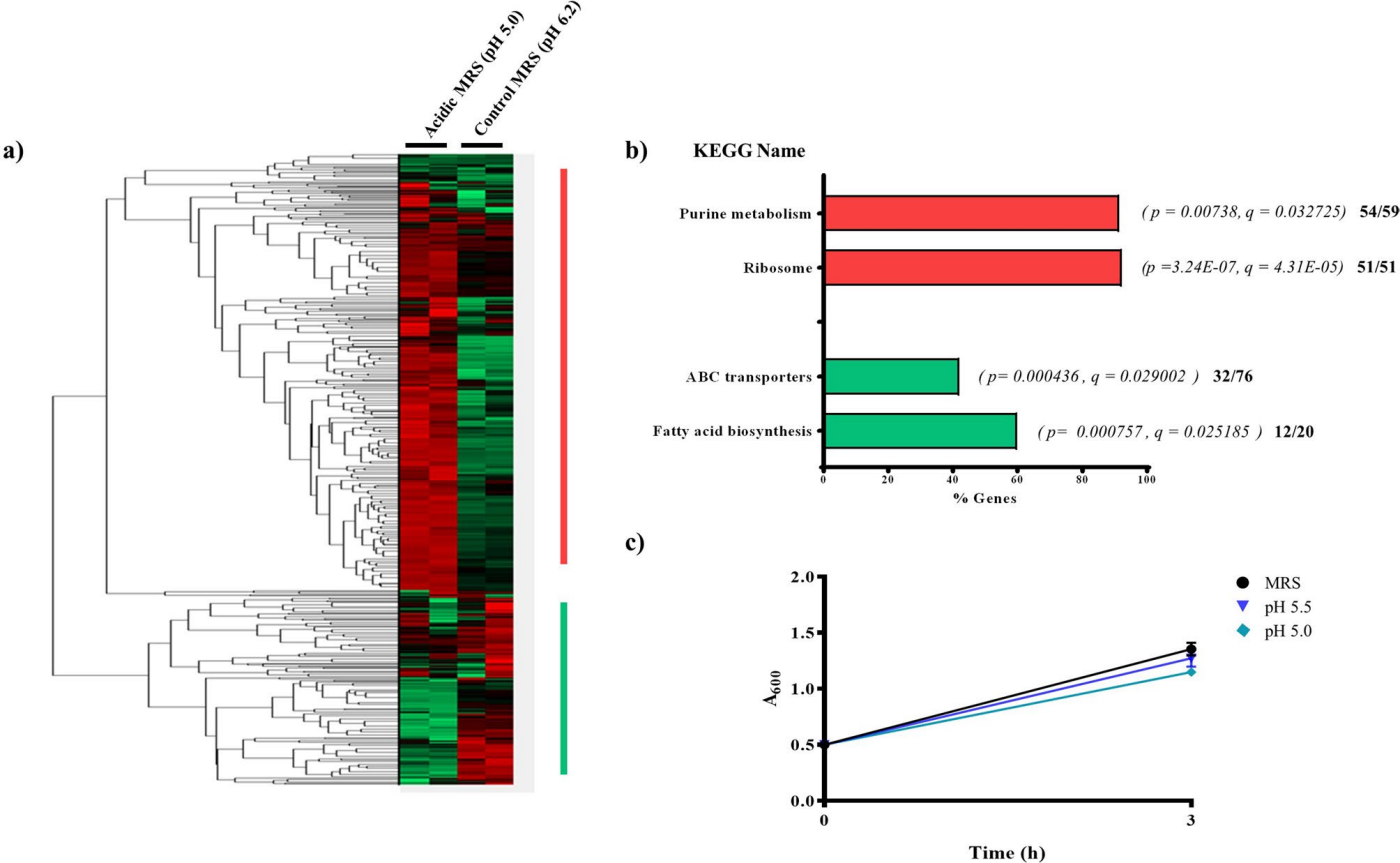
Transcriptome data analysis. The bacteria were cultivated for standard MRS (pH 6.2) and acidic MRS adjust pH 5.0 with lactic acid for 3 h, respectively. The heatmap was generated using the z-score value of the transcriptome (**a**). The categorically annotated Kyoto Encyclopedia of Genes and Genomes (KEGG) data have been presented as a bar graph, and the genes have been represented in the supplementary data. Genes by functional categories that were up-regulated (red) and down-regulated (green) in the acidic conditions (**b**). The growth of bacteria under acidic conditions was spectrophotometrically monitored at A600 nm (**c**). The brackets represent *p* and FDR (q) values and the genes are listed in Supplementary Table S1.

Transcriptome data analysis was performed using FPKM (Reads Per Kilobase Million) values of the processed data. The expression value was normalized to a z-score and hierarchial clustering was performed. By means of gene enrichment analysis, we determined several different GO or KEGG terms and metabolic pathways that were enriched with significantly up-regulated and down-regulated genes (Enrichment with ENSG option with q < 0.04 with Ben. Ho. FDR). The KEGG categorical enrichment showed an increase of purine and ribosomes, while fatty acid biosynthesis and ABC transport function were down-regulated (Fig. 1, Supplementary Table S1). Genes were considered differentially expressed when the pH was 5.0 vs. standard MRS (pH 6.2) and the Log2FC value was > 1 or <  − 1. DEG analysis demonstrated that gene expression increased for 25 genes and decreased for 13 genes (Table 1).

**Table 1:** Genes differentially expressed at the transcriptional level under acidic conditions.

Gene Name	log2FC	p-value	FDR	Description	ENSG gene ID	Protein name	eggNOG.ID	KEGG.ID
*lp_0018*	1.75	1.18E−27	3.00E−26	Oligopeptide ABC transporter	lp_0018	F9US48	COG4166	K02035
*cysE*	3.18	9.45E−87	1.47E−84	Serine acetyltransferase	lp_0254	F9UT52	COG1045	K00640
*cblB*	3.69	3.13E−95	5.15E−93	Cystathionine beta-lyase	lp_0255	F9UT53	COG0626	K01760
*cbs*	3.45	1.28E−70	1.41E−68	Cystathionine beta-synthase	lp_0256	F9UT54	COG0031	K01738
*pnuC1*	1.64	2.38E−218	8.95E−216	Nicotinamide mononucleotide transporter	lp_0259	F9UT56	COG3201	K03811
*treA*	1.20	0.00109	0.005036	Trehalose-6-phosphate hydrolase	lp_0263	F9UT60	COG0366	K01226
*pts4ABC*	1.38	2.08E−08	1.88E−07	PTS system, trehalose-specific IIBC component	lp_0264	F9UT61	COG2190	K02757
*lp_0302*	3.20	2.46E−64	2.09E−62	Extracellular transglycosylase	lp_0302	F9UTQ7	NOG102536	K01185
*lp_0304*	1.60	5.83E−32	1.85E−30	Extracellular transglycosylase	lp_0304	F9UTQ8	NOG62861	K01185
*lp_0783*	2.61	1.48E−116	3.01E−114	Oligopeptide ABC transporter	lp_0783	F9UM05	COG4166	K15580
*pyrD*	1.11	1.66E−29	4.61E−28	Dihydroorotate dehydrogenase	lp_2699	F9URI1	COG0167	K17828
*pyrAB*	1.19	2.37E−26	5.73E−25	Carbamoyl-phosphate synthase	lp_2700	F9URI2	COG0458	K01955
*pyrAA*	1.49	1.70E−30	5.17E−29	Carbamoyl-phosphate synthase	lp_2701	F9URI3	COG0505	K01956
*pyrC*	1.54	8.42E−30	2.39E−28	Dihydroorotase	lp_2702	F9URI4	COG0044	K01465
*pyrB*	1.65	8.95E−29	2.38E−27	Aspartate carbamoyltransferase	lp_2703	F9URI5	COG0540	K00609
*pyrR1*	1.16	5.56E−64	4.58E−62	Pyrimidine operon regulatory protein	lp_2704	F9URI6	COG2065	K02825
*lp_2809*	1.59	1.68E−29	4.62E−28	Extracellular protein	lp_2809	F9URS2	–	–
*lp_2810*	1.73	5.17E−13	7.14E−12	Glycosyl hydrolase	lp_2810	F9URS3	NOG68375	K07273
*lp_3014*	2.51	8.45E−15	1.31E−13	Extracellular transglycosylase	lp_3014	F9USE1	NOG62861	K18718
*lp_3050*	1.99	7.77E−02	2.08E−01	Extracellular transglycosylase	lp_3050	F9USH2	NOG102536	K01238
*copA*	1.23	0.05242	0.150308	Copper transporting ATPase	lp_3055	F9USH5	NOG150102	–
*lp_3177*	1.09	0.007399	0.028166	Hypothetical membrane protein	lp_3177	F9UT90	COG2217	K17686
*lp_3178*	1.20	0.004248	0.017189	Extracellular protein	lp_3178	F9UT91	NOG111565	–
*copB*	2.51	2.27E−42	1.09E−40	Copper transporting ATPase	lp_3363	F9UU49	COG2217	K01533
*lp_3421*	2.86	9.32E−271	6.15E−268	gamma-D-glutamate-meso-diaminopimelate muropeptidase	lp_3421	F9UUA0	COG0791	K18718
*mapB*	− 1.50	3.68E−08	3.19E−07	Maltose phosphorylase	lp_0181	F9USZ3	COG1554	K00691
*mtsC*	− 1.25	0.000367	0.001885	Manganese ABC transporter	lp_1095	F9UMQ9	COG1121	K11706
*aroI*	− 1.07	0.368778	0.641246	Shikimate kinase	lp_2033	F9UPZ0	COG0703	K00891
*cps4E*	− 1.34	0.128582	0.307524	Polysaccharide biosynthesis	lp_2104	F9UQ51	COG2148	K03606
*araT2*	− 1.36	3.09E−10	3.38E−09	Aminotransferase	lp_2684	F9URG9	COG0436	K00841
*livA*	− 1.24	0.10631	0.266582	Branched-chain amino acid ABC transporter	lp_2985	F9USB7	COG0683	K01999
*mntH3*	− 2.38	9.35E−223	4.11E−220	Manganese transport protein	lp_2992	F9USC2	COG1914	K03322
*lp_2993*	− 2.35	3.11E−243	1.64E−240	Nucleotide-binding protein	lp_2993	F9USC3	COG0589	–
*lrgA*	− 1.15	6.03E−06	4.09E−05	Murein hydrolase export protein	lp_3254	F9UTF1	NOG101785	K05338
*purA*	− 1.48	3.87E−31	1.20E−29	Adenylosuccinate synthetase	lp_3270	F9UTG3	COG0104	K01939
*guaC*	− 1.88	1.04E−41	4.67E−40	GMP reductase	lp_3271	F9UTG4	COG0516	K00364
*lp_3278*	− 1.47	0.000514	0.002559	Amino acid transport protein	lp_3278	F9UTG8	COG0531	K03294
*dapA2*	− 1.59	6.43E−19	1.18E−17	hydroxy-tetrahydrodipicolinate synthase	lp_2685	F9URH0	COG0329	K01714

Fold change in gene expression of group exposed to acidic conditions was calculated by edgeR R package. The minus symbol signifies down-regulation.

**Figure 2:**
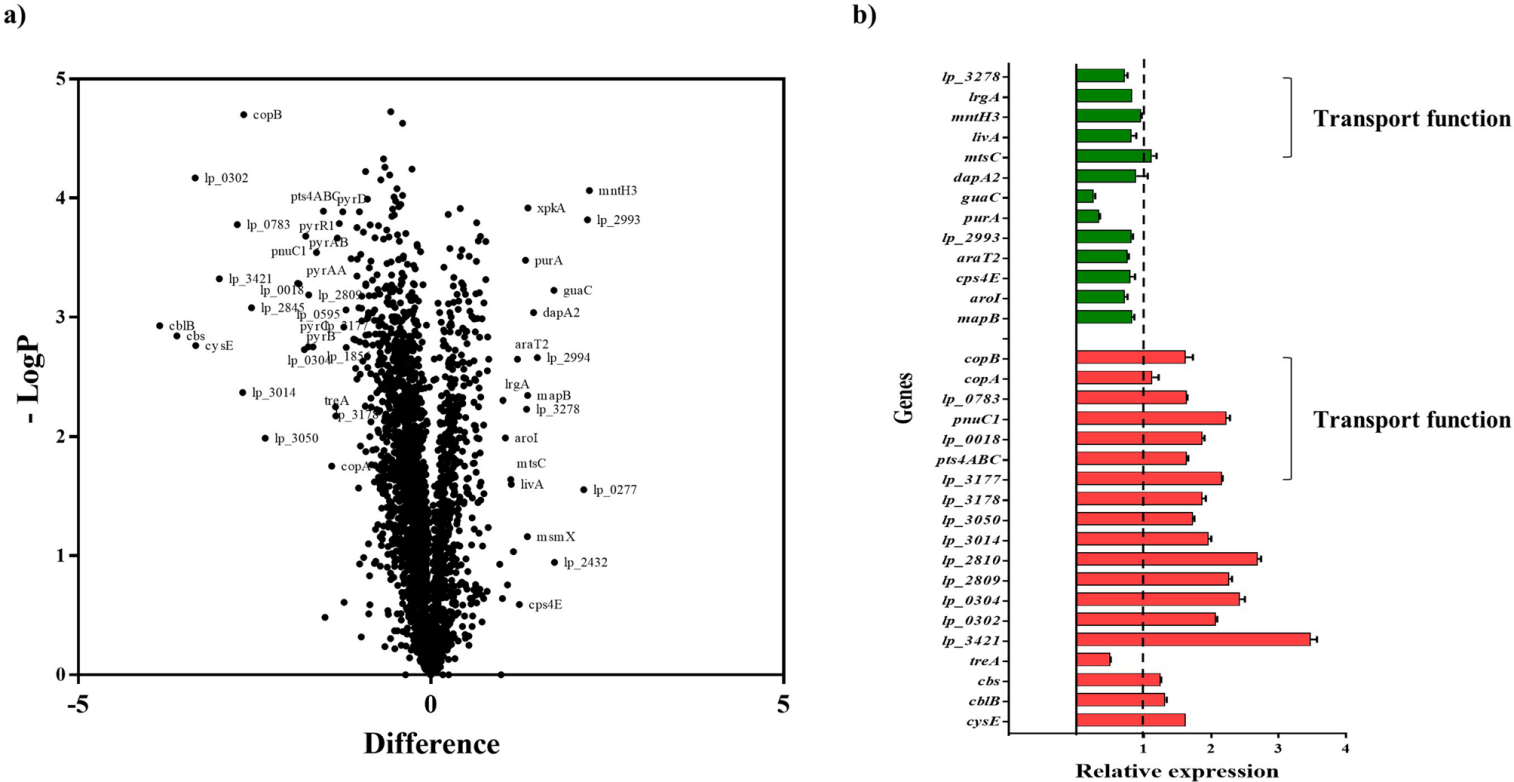
Volcano plot of differentially expressed genes under acidic conditions and qRT-PCR quantification. The volcano plot was generated with the difference in gene expression vs. the *p*-value (**a**). Gene expression was quantified using real-time RT-PCR. The relative expression was calculated by ΔΔC_T_ method (**b**). The red and green bars represent the up-regulated and down-regulated genes, respectively.

The following genes with amine biosynthetic functions were significantly up-regulated: alanine, aspartate metabolism (*pyrAA*), and amino acid metabolism-related genes (*cblB*, *cbs*, *cysE*); PTS system genes (*pts4ABC*); extracellular transglycosylase genes (*lp_0302*, *lp_0304*, *lp_3014*, *lp_3050*); oligo-peptide ABC transporter genes (*lp_0018*, *lp_0783*); nicotinamide nucleotide transporter genes (*pnuC1*); copper transporting ATPase genes (*copA*, *copB*); and carbamoyl phosphatase genes (*pyrAB*, *pyrAA*, *pyrC*). Genes denoting maltose phosphorylase (*mapB*), manganese transport (*mntH3*), amino acid transport protein (*lp_32*78), amine transport, ABC transporter (*livA*), manganese ABC transporter (*mtsC*), and glycosylphosphotransferase (*cps4E*) were significantly down-regulated (Table 1). The results of transcriptome analysis were validated using quantitative RT-PCR (Fig. 2). The KEGG functional category was annotated into the data and deposited in NCBI (GEO series accession number GSE143834).

### Effect of transport inhibitor on bacterial growth in acidic conditions

**Figure 3:**
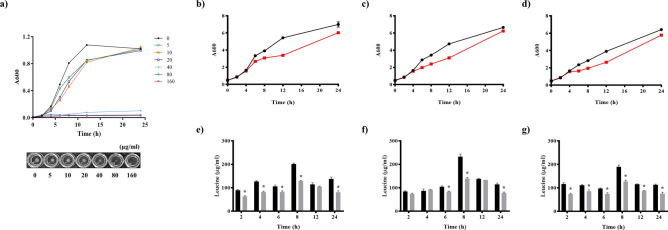
Effect of valinomycin on *L. plantarum* growth and intracellular leucine content under different acidic conditions. The effect of valinomycin on the growth of *L. plantarum* was measured spectrophotometrically at 600 nm and visibly monitored (**a**). *L. plantarum* was cultivated with 0, 5, 10, 20, 40, 80, or 160 μg/ml valinomycin. The *L. plantarum* growth and intracellular leucine content was determined at pH 6.2 (**b**,**e**), pH 5.5 (**c**,**f**), and pH 5.0 (**d**,**g**), respectively. The red line and grey bar indicate the presence of 10 µg/ml valinomycin (*p < 0.05).

In order to investigate its transport function, *L. plantarum* growth was monitored and compared between different acidic conditions ranging from pH 6.2 to 5.0. The ABC transport inhibitor valinomycin was added to the culture and bacterial growth was monitored. Valinomycin has been reported to inhibit ABC-type transport in bacteria^23^. *L. plantarum* growth was reduced by addition of 5–20 µg/ml of valinomycin and inhibited by 40 µg/ml of valinomycin (Fig. 3a). To evaluate the effect of acidity on bacterial nutrient uptake, the intracellular leucine content was monitored at different pH values in accordance with bacterial growth with 10 µg/ml of valinomycin. Leucine is an auxotrophic amino acid for *L. plantarum*, and it is known to be transported by ABC-type transport into the intracellular space^24–26^.

*Lactobacillus plantarum* growth was mostly affected by the addition of valinomycin. However, growth was restored at the later growth stage (Fig. 3b–d). The intracellular leucine content was at its maximum at 8 h of culture (189.3 ± 7.5–232.7 ± 11.0 µg/ml), and was found to be 232.7 ± 11.0 µg/ml at pH 5.5. This concentration is 201.0 ± 4.0 µg/ml in MRS or 189.3 ± 7.5 µg/ml at pH 5.0, despite higher growth in MRS than at pH 5.5. The addition of valinomycin significantly decreased the intracellular leucine content at all pH ranges (*p* < 0.05).

**Figure 4:**
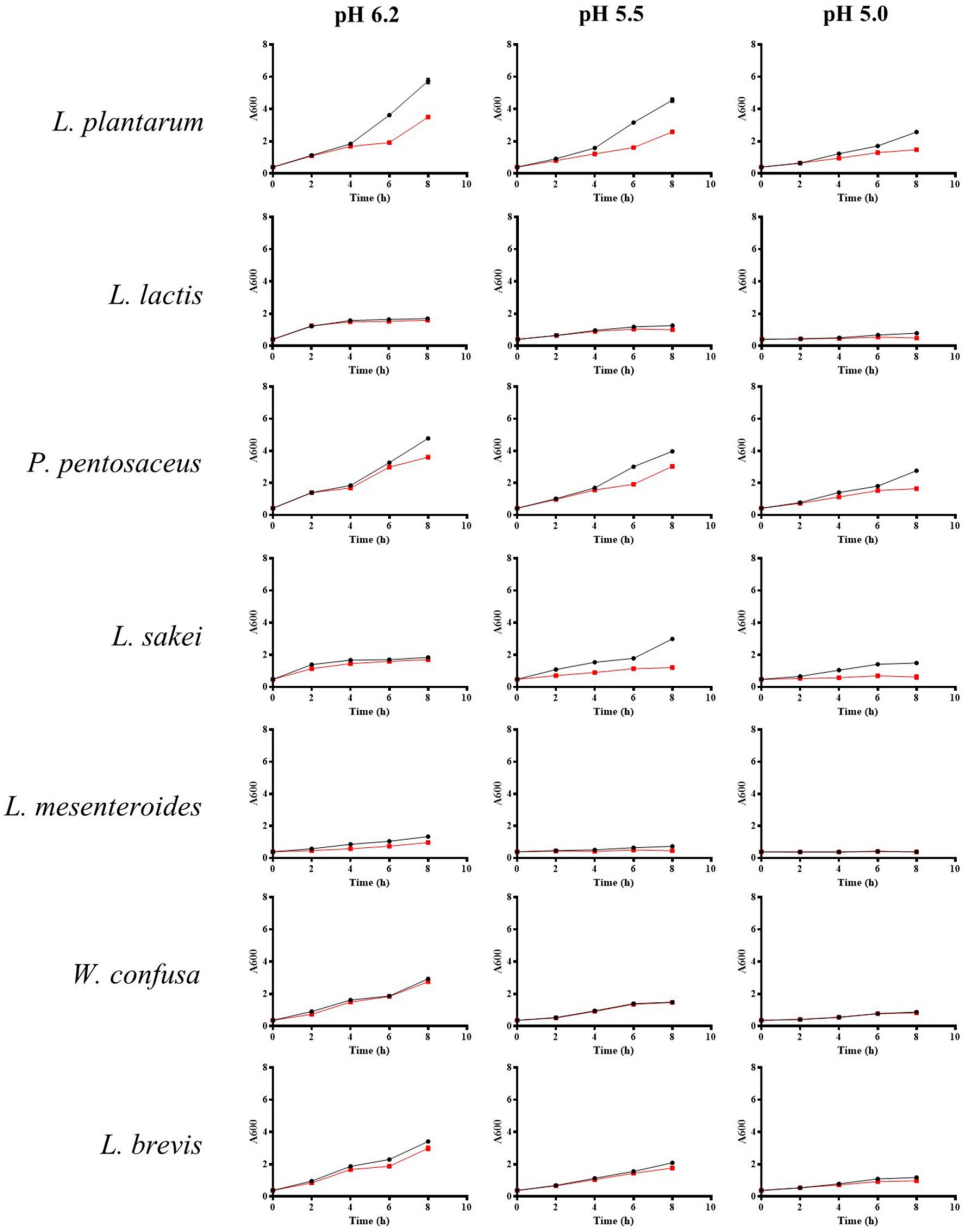
Lactic acid bacteria growth under different acidic conditions in the presence of valinomycin. Bacterial was cultivated in MRS media, and the pH was adjusted with lactic acid. Bacterial growth was monitored spectrophotometrically at 600 nm. The red line indicates the addition of valinomycin to the culture (10 µg/ml).

Valinomycin addition decreased the intracellular leucine content by 23–40%. In MRS (pH 6.2), the leucine content was decreased by 23.2% at 6 h and 35.5% at 8 h. Minimum intracellular leucine inhibition was detected in media with pH 5.5, with a 12.3% and 20.5% decrease detected at 2 h and 6 h, respectively (Fig. 3e–g). This inhibition was lower than that in MRS or pH 5.0 media. Next, we compared the growth inhibition between other LAB. Bacterial growth and inhibition upon valinomycin treatment was compared with *Lactobacillus brevis*, *Pediococcus pentosaceus*, *Leuconostoc lactis*, *Lactobacillus sakei*, *Leuconostoc mesenteroides*, and *Weissella confusa* and showed the growth reduction at 5–20 µg/ml and the growth inhibition at 40 µg/ml concentration (Supplementary Fig. S1). Interestingly, the LAB showed different degrees of inhibition in the presence of valinomycin. There was no difference in *L. brevis* and *W. confusa* growth in the presence of valinomycin. Interestingly*,* the growth of *L. sakei* was slightly inhibited by valinomycin at a lower pH (pH 5.5–5.0) (Fig. 4).

## Discussion

*Lactobacillus plantarum* is a facultative hetero-fermentative gram-positive bacterium. It is an extremely versatile LAB, and has been isolated from the gastrointestinal tract, meat, fish, raw and fermented products^27^. *L. plantarum* has one of the largest genomes among LAB, but analysis of its genome sequence has demonstrated that it does not show evolved traits specializing the gene for specific environments^28^.

An acidic environment is the most common element observed during fermentation. This contributes to inhibition of undesired bacterial growth, which is usually pathogenic and disrupts fermentation. However, acidic conditions also affect the adaptation of LAB. For example, *Lactobacillus rhamnosus* GG modulates its pyruvate metabolism depending on the growth pH, and possesses amino acid transport and adhesion-related genes^29^.

Previous report showed that the pH changes from pH 6 to pH 4 during the fourth week of fermentation. The pH in the kimchi was dramatically decreased at the first week, as it lowered from pH 6 to pH 5 due to the sharp increase of the LAB^5,30^. So this periods are important for the bacterial adaption.

In this study, we found that acidic conditions (pH 5) affected the expression of genes located in the functional categories of purine metabolism, ribosomes, fatty acid biosynthesis, and ABC transport (Fig. 1). DEG analysis showed that specific genes were significantly up- (FC > 1) or down-regulated (FC < 1) (Table 1). Several genes involved in carbohydrate metabolism, especially transglycosylase genes, were up-regulated. Previous studies have reported that alteration of carbohydrate metabolism under acid stress conditions enables bacteria to better resist acid stress by increasing their energy supply^31^.

Phosphotransferase system (PTS) genes were observed to be up-regulated under conditions of oxidative stress, such as upon H_2_O_2_ or hydroxybenzoic acid exposure^20^. Interestingly, copper export ATPase gene (*copA*, *copB*) expression was increased. Previous research has shown that under oxidative stress, the copper export ATPase (*copB*) gene of *L. plantarum* is inactivated. This enhances its sensitivity to the oxidative stress conditions induced by H_2_O_2_ and Cu^+^ or Cu^2+^ participating in essential redox reactions^32^.

The expression and transport activity of ABC transporters are tightly regulated to balance the need for essential nutrients. Expression of DalS, the substrate binding protein (SBP) of the d-alanine ABC transporter (STM1633–STM1636) in *Salmonella typhimurium*, limits exposure to oxidative damage by d-amino acid oxidase in neutrophils^33^. Increased ABC-type oligopeptide transport proteins (*opp*) are associated with starvation conditions due to transport changes. *Opp* function as sensors for environmental change genes involved in amino acid biosynthesis and transport, thus counteracting amino acid starvation^34^. These results show that much of the gene expression observed under acidic conditions is similar to that observed under oxidative stress conditions.

Altogether, bacterial transport was mostly affected by acid stress conditions. Five of the 13 significantly down-regulated genes and 7 of the 25 significantly up-regulated genes possess transport-related functions (Table 1). These results suggest that acidic conditions affect bacterial adaptation, particularly regarding nutrient transport functions.

Figure 3 shows the growth inhibition of valinomycin in culture. Valinomycin effectively inhibited *L. plantarum* growth even under acidic conditions, indicating that transport functions were active under these conditions.

We previously identified that *L. plantarum* intracellular leucine and leucine metabolite content was associated with acidity^5^. The leucine biosynthesis pathways were found to be absent in all *L. plantarum *strains, as reported in previous studies^35^, and severe growth inhibition was observed in its absence^26^. Additionally, it was reported that leucine was transported via the ABC-type transporter^36^. We therefore quantified the intracellular leucine content of bacteria grown at different pH ranges (Fig. 3).

Although higher bacterial growth was observed in standard MRS (pH 6.2) media, the intracellular leucine content was higher in pH 5.5 culture. This indicates that the optimal bacterial leucine transport could be controlled by acidity during fermentation, and that transport function is most active at approximately pH 5.5. This pH range corresponds to the initial stage of kimchi fermentation (weeks 1–2 of fermentation). Next, we compared the inhibition of the diverse LAB originating from kimchi by valinomycin (Fig. 4).

*Lactobacillus plantarum*, *P. pentosaceus*, *L. sakei*, and *L. mesenteroides* growth was inhibited by valinomycin, while *L. brevis*, *W. confusa* and *L. lactis* did not demonstrate any difference in growth. The growth of *L. sakei* was slightly inhibited under acidic conditions of around pH 5.5 and 5.0. These results indicate that the transport function of *L. plantarum* is active under a wide range of acidic conditions.

Lactic acid easily diffuses into the cytoplasm and dissociates into protons and anions, disrupts the intracellular pH, and impairs normal cellular function^31^. During fermentation, *Lactobacillus* transports lactic acid outside the cell as lactate ions via an electrogenic proton-lactate symporter^34^. In this study, we demonstrated that *L. plantarum* had a wide range of active transport and acidification functions and modulates leucine uptake. The adaptation of LAB to acidic conditions is a fundamental element of successful fermentation. Interspecies metabolite exchange occurs via nutrient cross feeding. This trophic interaction enables multiple groups of organisms to survive on limited nutrient sources, increasing community density^37^. The differences in nutrient transport activity among LAB may affect bacterial survival under certain circumstances. Several studies on the stress response of *L. plantarum* showed higher up- and down-regulation of transport functions in diverse stress conditions induced by resveratrol, hydrogen peroxide, or *p*-coumaric acid^19–21^. The transport function may, therefore, be a crucial component for adaptation under diverse stress conditions.

These results also suggested that modulating the transport function could be pivotal to controlling fermentation via bacterial growth in the relevant industries.

## Materials and methods

### Bacterial strain and culture

A bacterial strain was previously isolated from kimchi and identified previously^5,8,38,39^. *Lactobacillus plantarum* wikim18, *Leuconostoc lactis* WiKim48, *Pediococcus pentosaceus* WiKim20, *Lactobacillus sakei* WiKim49, *Leuconostoc mesenteroides* WiKim19, *Weissella confusa* WiKim29, and *Lactobacillus brevis* WiKim47 were used in this experiment.

The bacteria were cultivated at 30 °C for 12 h in De Man, Rogosa and Sharpe (MRS) media (Miller, Becton Dickinson, and Co., Sparks, MD, USA). The bacterial culture was harvested by centrifugation at 5000×*g* for 20 min and diluted to into MRS media by absorbance of 0.5 at 600 nm. To monitor the growth effect in the different pH, MRS was prepared with desired pH with addition of lactic acid. The valinomycin (Sigma-Aldrich, St. Louis, MO, USA) was added into the MRS media at the final concentration of 10 μg/ml. The effect of valinomycin on growth of LAB was measured at 600 nm using microplate reader (Tecan, mannedorf, Zurich, Switzerland).

### Determination of the minimum inhibitory concentration (MIC)

MIC was measured using twofold serial dilutions methods^40^. The bacteria was cultivated in MRS media and adjusted to absorbance of 0.01 at 600 nm. The 100 μl of serial diluted valinomycin (from 160 to 5 μg/ml) were added to 100 μl of bacterial suspension in 96 well-plates and incubated at 30 °C for 24 h. The effect of valinomycin concentration on the bacterial growth was measured at 600 nm using microplate reader (Tecan) and visible growth was monitored after 12 h at 30 °C.

### RNA isolation and transcriptome analysis

RNA sequencing was performed at Chunlab (Seoul, Korea). To evaluate the global gene expression of *L. plantarum* in acidic conditions, the bacterial culture was harvested by centrifugation at 5000×*g* for 20 min and diluted with acidic MRS adjusted pH with lactic acid (pH 5.0) and standard MRS (pH 6.2) by optical absorbance of 0.5 at 600 nm and further incubated at 30 °C for 3 h. Total RNA was extracted using RNeasy Mini kits (Qiagen, USA) as per the manufacturer’s instructions. The isolated RNA was stored at − 80 °C until use. The Ribo‐Zero rRNA removal kit (Epicentre, USA) was used for ribosomal RNA depletion according to the manufacturer’s instructions. Libraries for Illumina sequencing were made with the TruSeq Stranded mRNA sample prep kit (Illumina, USA) according to the manufacturer’s instructions. RNA sequencing was performed on the Illumina HiSeq 2500 platform using single‐end 50 bp sequencing. bcl2fastq v1.8.4 software combines per-cycle BCL basecall files generated by Illumina sequencing instruments, translating them into FASTQ files. The raw FASTQ files were split into files containing about 20,000,000 reads and checked for quality using the FASTQC (v0.11.7). The reads were filtered (removing sequences that did not pass Illumina’s quality filter) and trimmed based on the quality results by trimmomatic-0.36. Quality filtered reads were aligned to the reference genome sequence using Bowtie2 (v2.2.3). The sequence data for the reference genome was retrieved from the NCBI database (GCF_000203855.3, *L. plantarum* WCFS1). Quality filtered reads were aligned to the reference genome sequence using Bowtie2.

### Transcriptome data analysis and deposit

The relative transcript-x abundance was measured in fragments in reads per kilobase of exon sequence per million mapped sequence reads (FPKM). The evolutionary genealogy of genes: Non-supervised Orthologous Groups (eggNOG) database was used to cluster genes into functionally related groups, and the Kyoto Encyclopedia of Genes and Genomes (KEGG) database was used to analyze metabolic pathways. The results of mapping and differentially expressed gene (DEG) analysis were visualized using Perseus^41,42^. Functional annotation was performed using DAVID (DAVID Bioinformatics Resources 6.8)^43^. DEGs were calculated as log2FC using edgeR in R package^44^. The RNA-seq data discussed in this publication have been deposited in NCBI’s Gene Expression Omnibus and are accessible through GEO Series accession number GSE143834 (https://www.ncbi.nlm.nih.gov/geo/query/acc.cgi?acc=GSE143834).

### Quantitative RT-PCR

Real-time RT-PCR was performed to validate the transcriptome analysis data. The gene expression levels were measured using quantitative real-time PCR. The bacteria were cultivated for a further 3 h in MRS media, and pH was adjusted to 5.0 with lactic acid. The bacterial RNA was extracted using Trizol (Invitrogen, CA, USA) according to the manufacturer’s instructions. Approximately 1 µg RNA was reverse transcribed, cDNA was generated, and RT-PCR was performed using SYBR green premix (Bio-Rad, Hercules, CA, USA). The relative expression level was calculated and normalized to that of the *16S rRNA* gene^5^. Primers were designed based on nucleotide sequences from *L. plantarum* of NCBI database (GCF_000203855.3, *L. plantarum* WCFS1) (Supplementary Table S2).

### Quantification of intracellular leucine content

We monitored the effect of pH changes on amino acid transport and the intracellular leucine content using LC–MS/MS technology. A TripleTOF 5600 plus instrument (SCIEX, Framingham, MA, USA) coupled with an Acquity UPLC system (Waters, Milford, MA, USA) was used to characterize the metabolites and quantify the intracellular and extracellular leucine content of bacteria. Leucine was quantified in negative MRM mode using the following transitions: Leucine, m/z 130 > 130; Salicin, m/z 285 > 123. A reversed-phase column (Acquity UPLC BEH C18 column 2.1 × 100 mm, 1.7 μm particle size; Waters) was used to separate the compounds. The mobile phase consisted of distilled water (solvent A) and acetonitrile (solvent B) containing 10 mM ammonium acetate at a flow rate of 0.4 ml/min. All experiments were performed in triplicate. The data are presented as means and standard derivations. A two-way analysis of variance test was performed using GraphPad Prism v7 software with the Tukey’s multiple comparisons test.

## Supplementary information

Supplementary Information.
