# Supplementary material for: Characterization of transcriptional response of Lactobacillus plantarum under acidic conditions provides insight into bacterial adaptation in fermentative environments
Source: Sci Rep. 2020 Nov 5;10:19203. doi: 10.1038/s41598-020-76171-6 (PMC7645587; doi:10.1038/s41598-020-76171-6)
Supplement: Supplementary file 1 — Supplementary Information. [file 41598_2020_76171_MOESM1_ESM.docx]

**Supplementary Information**

**Characterization of transcriptional response of *Lactobacillus plantarum* under acidic conditions provides insight into bacterial adaptation in fermentative environments**

**Sera Jung and Jong-Hee Lee***

Advanced Process Technology and Fermentation Research Group, Research and Development Division, World Institute of Kimchi, Gwangju, 61755, Republic of Korea

*Corresponding author:

Jong-Hee Lee, Ph.D.

Advanced Process Technology and Fermentation Research Group

Research and Development Division, World Institute of Kimchi, Gwangju, 61755, Republic of Korea

Tel.: + 82-62-610-1730, Fax: +82-62-610-1853

E-mail: leejonghee@wikim.re.kr


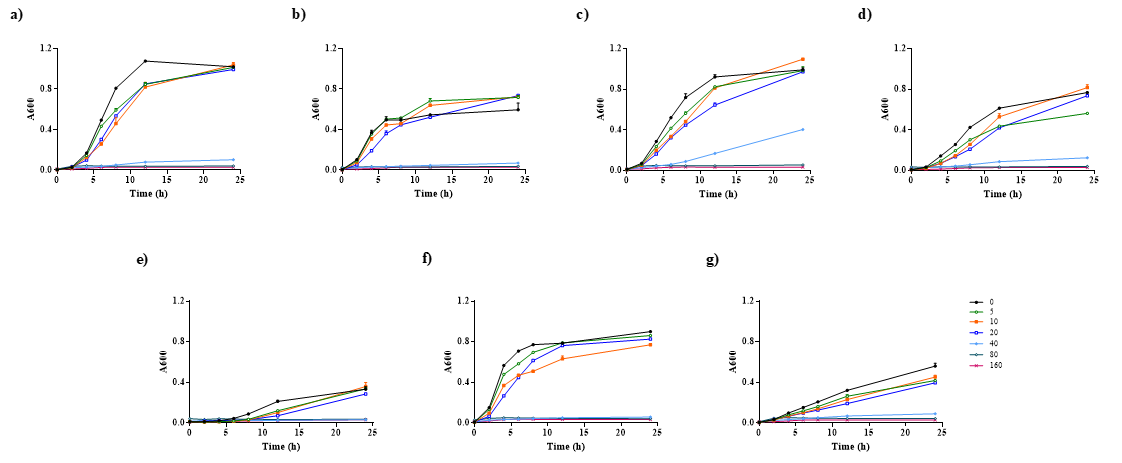


**Supplementary Figure S1. The effect of valinomycin on lactic acid bacteria growth.** The growth of lactic acid bacteria was measured spectrophotometrically at 600 nm with different concentration (0, 5, 10, 20, 40, 80, or 160 μg/ml) of valinomycin. a) *L. plantarum,* b) *L. lactis*, c) *P. pentosaceus,* d) *L. sakei,* e) *L. mesenteroides,* f) *W. confusa,* g) *L.brevis*.

**Supplementary Table S1. KEGG categorical annotated genes.**

| **KEGG Name (genes in cluster/total genes)** | **KEGG ID** | | **Gene Name** | **ENSG Gene ID** | **Protein Name** | | **Description** | |
| --- | --- | --- | --- | --- | --- | --- | --- | --- |
| Purine metabolism  (54 genes/59 genes) | K00364 | | *guaC* | lp_3271 | F9UTG4;Q88SV5 | | GMP reductase | |
|  | K01939 | | *purA* | lp_3270 | F9UTG3;Q88SV6 | | Adenylosuccinate synthetase | |
|  | K01756 | | *purB* | lp_3269 | F9UTG2 | | Adenylosuccinate lyase | |
|  | K01588 | | *purE* | lp_2729 | F9URK5 | | N5-carboxyaminoimidazole ribonucleotide mutase | |
|  | K01589 | | *purK1* | lp_2728 | F9URK4 | | N5-carboxyaminoimidazole ribonucleotide synthase | |
|  | K01923 | | *purC* | lp_2727 | F9URK3;Q88U22 | | Phosphoribosylaminoimidazole-succinocarboxamide synthase | |
|  | K01952 | | *purS* | lp_2726 | F9URK2 | | Phosphoribosylformylglycinamidine synthase subunit | |
|  | K01952 | | *purQ* | lp_2725 | F9URK1;Q88U24 | | Phosphoribosylformylglycinamidine synthase subunit | |
|  | K01952 | | *purL* | lp_2724 | F9URK0;Q88U25 | | Phosphoribosylformylglycinamidine synthase subunit | |
|  | K00764 | | *purF* | lp_2723 | F9URJ9 | | Amidophosphoribosyltransferase | |
|  | K01933 | | *purM* | lp_2722 | F9URJ8 | | Phosphoribosylformylglycinamidine cyclo-ligase | |
|  | K11175 | | *purN* | lp_2721 | F9URJ7 | | Phosphoribosylglycinamide formyltransferase | |
|  | K00602 | | *purH* | lp_2720 | F9URJ6;Q88U29 | | Bifunctional purine biosynthesis protein | |
|  | K01945 | | *purD* | lp_2719 | F9URJ5;Q88U30 | | Phosphoribosylamine-glycine ligase | |
|  | K00759 | | *lp_1289* | lp_1289 | F9UN69 | | Purine/pyrimidine phosphoribosyltransferase | |
|  | K10353 | | *dgk2* | lp_1329 | F9UNA0 | | Deoxynucleoside kinase | |
|  | K00942 | | *gmk2* | lp_0866 | F9UM77 | | Guanylate kinase | |
|  | K00527 | | *nrdD* | lp_2932 | F9US18 | | Anaerobic ribonucleoside-triphosphate reductase | |
|  | K00939 | | *adk* | lp_1058 | F9UMM6;Q88XW5 | | Adenylate kinase | |
|  | K00525 | | *nrdE* | lp_0693 | F9ULT0 | | Ribonucleoside-diphosphate reductase | |
|  | K00526 | | *nrdF* | lp_0692 | F9ULS9 | | Ribonucleoside-diphosphate reductase | |
|  | K00948 | | *prs2* | lp_2166 | F9UQA2;Q88VA5 | | Putative ribose-phosphate pyrophosphokinase | |
|  | K03040 | | *rpoA* | lp_1062 | F9UMN1;Q88XW0 | | DNA-directed RNA polymerase subunit alpha | |
|  | K02341 | | *holB* | lp_0705 | F9ULU0 | | DNA-directed DNA polymerase III, delta' subunit | |
|  | K02340 | | *holA* | lp_2128 | F9UQ72 | | DNA-directed DNA polymerase III, delta chain | |
|  | K01524 | | *ppx3* | lp_0843 | F9UM58 | | Exopolyphosphatase | |
|  | K12700 | | *lp_0363* | lp_0363 | F9UTW0 | | Purine nucleosidase | |
|  | K01239 | | *lp_2591* | lp_2591 | F9URA0 | | Purine nucleosidase | |
|  | K00760 | | *hprT* | lp_0546 | F9UL19 | | Hypoxanthine phosphoribosyltransferase | |
|  | K01120 | | *lp_3251* | lp_3251 | F9UTE9 | | 3',5'-cyclic-nucleotide phosphodiesterase | |
|  | K01951 | | *guaA* | lp_0914 | F9UMB5;Q88Y74 | | GMP synthase | |
|  | K01524 | | *ppx1* | lp_0446 | F9UU31 | | Exopolyphosphatase | |
|  | K01835 | | *pgm* | lp_0764 | F9ULZ0 | | Phosphoglucomutase | |
|  | K01524 | | *ppx2* | lp_0841 | F9UM56 | | Exopolyphosphatase | |
|  | K03048 | | *rpoE* | lp_0480 | F9UKW2;Q88Z77 | | Probable DNA-directed RNA polymerase subunit delta | |
|  | K10353 | | *dgk1* | lp_1011 | F9UMJ3 | | Deoxyadenosine kinase | |
|  | K03046 | | *rpoC* | lp_1022 | F9UMJ9;Q88XZ2 | | DNA-directed RNA polymerase subunit beta | |
|  | K01589 | | *purK2* | lp_1141 | F9UMU4 | | Formate-dependent phosphoribosylglycinamide formyltransferase | |
|  | K03763 | | *polC* | lp_2045 | F9UQ02;Q88VK2 | | DNA polymerase III PolC-type | |
|  | K02343 | | *dnaX* | lp_0698 | F9ULT4 | | DNA polymerase III subunit gamma | |
|  | K03043 | | *rpoB* | lp_1021 | F9UMJ8;Q88XZ3 | | DNA-directed RNA polymerase subunit beta | |
|  | K07816 | | *lp_2223* | lp_2223 | F9UQE7 | | GTP pyrophosphokinase | |
|  | K01529 | | *lp_1790* | lp_1790 | F9UPD8 | | Phosphohydrolase | |
|  | K00873 | | *pyk* | lp_1897 | F9UPM3 | | Pyruvate kinase | |
|  | K01515 | | *lp_2762* | lp_2762 | F9URN3 | | Phosphohydrolase | |
|  | K02342 | | *lp_0811* | lp_0811 | F9UM30 | | DNA-directed DNA polymerase III, epsilon chain | |
|  | K02338 | | *dnaN* | lp_0002 | F9US34 | | Beta sliding clamp | |
|  | K01239 | | *lp_2825* | lp_2825 | F9URT3 | | Purine nucleosidase | |
|  | K00759 | | *apt* | lp_2086 | F9UQ35;Q88VH0 | | Adenine phosphoribosyltransferase | |
|  | K00951 | | *relA* | lp_1987 | F9UPV0 | | GTP pyrophosphokinase | |
|  | K03060 | | *rpoZ* | lp_1613 | F9UNY7;Q88WL6 | | DNA-directed RNA polymerase subunit omega | |
|  | K03816 | | *xpt* | lp_1139 | F9UMU2;Q88XQ4 | | Xanthine phosphoribosyltransferase | |
|  | K00948 | | *prs1* | lp_0471 | F9UKV5;Q88Z84 | | Ribose-phosphate pyrophosphokinase 1 | |
|  | K00951 | | *lp_0293* | lp_0293 | F9UTP8 | | GTP pyrophosphokinase | |
| Ribosome (51 genes/ 51 genes) | | K02935 | | *rplL* | | lp_0622 | F9UL87 | Cytochrome c oxidase subunit 1 |
|  |  | K02864 | | *rplJ* | | lp_0621 | F9UL86 | 50S ribosomal protein L10 |
|  |  | K02933 | | *rplF* | | lp_1051 | F9UMM0 | 50S ribosomal protein L6 |
|  |  | K02881 | | *rplR* | | lp_1052 | F9UMM1 | 50S ribosomal protein L18 |
|  |  | K02907 | | *rpmD* | | lp_1054 | F9UMM3 | 50S ribosomal protein L30 |
|  |  | K02988 | | *rpsE* | | lp_1053 | F9UMM2 | 30S ribosomal protein S5 |
|  |  | K02876 | | *rplO* | | lp_1055 | Q88XW7 | 50S ribosomal protein L15 |
|  |  | K02954 | | *rpsN* | | lp_2216 | F9UQE0 | 30S ribosomal protein S14 |
|  |  | K02879 | | *rplQ* | | lp_1063 | F9UMN2 | 50S ribosomal protein L17 |
|  |  | K02994 | | *rpsH* | | lp_1050 | F9UML9 | 30S ribosomal protein S8 |
|  |  | K02931 | | *rplE* | | lp_1047 | F9UML7 | 50S ribosomal protein L5 |
|  |  | K02895 | | *rplX* | | lp_1046 | F9UML6 | 50S ribosomal protein L5 |
|  |  | K02961 | | *rpsQ* | | lp_1044 | F9UML4 | 30S ribosomal protein S17 |
|  |  | K02878 | | *rplP* | | lp_1041 | F9UML2 | 50S ribosomal protein L16 |
|  |  | K02982 | | *rpsC* | | lp_1040 | F9UML1 | 30S ribosomal protein S3 |
|  |  | K02890 | | *rplV* | | lp_1039 | F9UML0 | 50S ribosomal protein L22 |
|  |  | K02911 | | *rpmF* | | lp_1535a | F9UNS1 | 50S ribosomal protein L32 |
|  |  | K02948 | | *rpsK* | | lp_1061 | F9UMN0 | 30S ribosomal protein S11 |
|  |  | K02863 | | *rplA* | | lp_0620 | F9UL85 | 50S ribosomal protein L1 |
|  |  | K02867 | | *rplK* | | lp_0619 | F9UL84 | 50S ribosomal protein L11 |
|  |  | K02902 | | *rpmB* | | lp_1624 | F9UNZ7 | 50S ribosomal protein L28 |
|  |  | K02919 | | *rpmJ* | | lp_1059a | F9UMM8 | 50S ribosomal protein L36 |
|  |  | K02952 | | *rpsM* | | lp_1060 | F9UMM9 | 30S ribosomal protein S13 |
|  |  | K02904 | | *rpmC* | | lp_1043 | F9UML3 | 50S ribosomal protein L29 |
|  |  | K02874 | | *rplN* | | lp_1045 | F9UML5 | 50S ribosomal protein L14 |
|  |  | K02871 | | *rplM* | | lp_1077 | F9UMP4 | 50S ribosomal protein L13 |
|  |  | K02886 | | *rplB* | | lp_1036 | F9UMK8 | 50S ribosomal protein L2 |
|  |  | K02926 | | *rplD* | | lp_1034 | F9UMK6 | 50S ribosomal protein L4 |
|  |  | K02990 | | *rpsF* | | lp_0009 | F9US39 | 30S ribosomal protein S6 |
|  |  | K02959 | | *rpsP* | | lp_1636 | F9UP08 | 30S ribosomal protein S16 |
|  |  | K02887 | | *rplT* | | lp_1517 | F9UNQ4 | 50S ribosomal protein L20 |
|  |  | K02916 | | *rpmI* | | lp_1516 | F9UNQ3 | 50S ribosomal protein L35 |
|  |  | K02965 | | *rpsS* | | lp_1038 | F9UMK9 | 30S ribosomal protein S19 |
|  |  | K02892 | | *rplW* | | lp_1035 | F9UMK7 | 50S ribosomal protein L23 |
|  |  | K02992 | | *rpsG* | | lp_1026 | F9UMK2 | 50S ribosomal protein L23 |
|  |  | K02946 | | *rpsJ* | | lp_1032 | F9UMK4 | 30S ribosomal protein S10 |
|  |  | K02963 | | *rpsR* | | lp_0011 | F9US41 | 30S ribosomal protein S18 |
|  |  | K02950 | | *rpsL* | | lp_1025 | F9UMK1 | 30S ribosomal protein S12 |
|  |  | K02968 | | *rpsT* | | lp_2126 | F9UQ71 | 30S ribosomal protein S20 |
|  |  | K02996 | | *rpsI* | | lp_1078 | F9UMP5 | 30S ribosomal protein S9 |
|  |  | K02906 | | *rplC* | | lp_1033 | F9UMK5 | 50S ribosomal protein L3 |
|  |  | K02967 | | *rpsB* | | lp_2055 | F9UQ10 | 30S ribosomal protein S2 |
|  |  | K02884 | | *rplS* | | lp_1640 | F9UP12 | 50S ribosomal protein L19 |
|  |  | K02986 | | *rpsD* | | lp_2331 | F9UQN6 | 30S ribosomal protein S4 |
|  |  | K02956 | | *rpsO* | | lp_2125 | F9UQ70 | 30S ribosomal protein S15 |
|  |  | K02913 | | *rpmG* | | lp_0615;lp_1569 | F9UL80 | 30S ribosomal protein S15 |
|  |  | K02970 | | *rpsU* | | lp_1973 | F9UPT7 | 30S ribosomal protein S21 |
|  |  | K02899 | | *rpmA* | | lp_1594 | F9UNW9 | 50S ribosomal protein L27 |
|  |  | K02939 | | *rplI* | | lp_0013 | F9US43 | 50S ribosomal protein L9 |
|  |  | K02945 | | *rpsA* | | lp_1882 | F9UPL0 | 30S ribosomal protein S1 |
|  |  | K02888 | | *rplU* | | lp_1592 | F9UNW7 | 50S ribosomal protein L21 |

**Supplementary Table S1. KEGG categorical annotated genes (cont.)**

| **KEGG Name (genes in cluster/total genes)** | **KEGG ID** | **Gene Name** | **ENSG Gene ID** | **Protein Name** | **Description** |
| --- | --- | --- | --- | --- | --- |
| ABC transporters (32 genes/76 total genes) | K02042 | *phnE2* | lp_0713 | F9ULU8 | Phosphonates ABC transporter |
|  | K05813 | *ugpB* | lp_1327 | F9UN98 | Glycerol-3-phosphate ABC transporter |
|  | K01990 | *lp_1945* | lp_1945 | F9UPR5 | Multidrug ABC transporter |
|  | K16787 | *lp_0149* | lp_0149 | F9USW5;Q88ZZ2 | Putative ABC transporter ATP-binding protein |
|  | K02010 | *lp_1750* | lp_1750 | F9UPA4 | Iron ABC transporter |
|  | K01999 | *livA* | lp_2985 | F9USB7 | Branched-chain amino acid ABC transporter |
|  | K16787 | *lp_0218* | lp_0218 | F9UT23 | ABC transporter |
|  | K05847 | *choQ* | lp_0368 | F9UTW5 | Glycine betaine/carnitine/choline ABC transporter |
|  | K02050 | *lp_2077* | lp_2077 | F9UQ28 | Nitrate/sulfonate/bicarbonate ABC transporter |
|  | K05846 | *choS* | lp_0367 | F9UTW4 | Glycine betaine/carnitine/choline ABC transporter |
|  | K02049 | *lp_2076* | lp_2076 | F9UQ27 | Nitrate/sulfonate/bicarbonate ABC transporter |
|  | K10112 | *msmX* | lp_0180 | F9USZ2 | Maltodextrin ABC transporter |
|  | K02042 | *phnE1* | lp_0712 | F9ULU7 | Phosphonates ABC transporter |
|  | K01998 | *livC* | lp_2983 | F9USB5 | Branched-chain amino acid ABC transporter |
|  | K02044 | *phnD* | lp_0715 | F9ULV0 | Phosphonates ABC transporter |
|  | K16785 | *lp_0148* | lp_0148 | F9USW4 | Cobalt ABC transporte |
|  | K10041 | *glnQ2* | lp_0878 | F9UM84 | Glutamine ABC transporter |
|  | K01992 | *lp_1944* | lp_1944 | F9UPR4 | Multidrug ABC transporter |
|  | K10039 | *glnH1* | lp_0881 | F9UM85 | Glutamine ABC transporter |
|  | K01995 | *livD* | lp_2982 | F9USB4 | Branched-chain amino acid ABC transporter |
|  | K16785 | *lp_0217* | lp_0217 | F9UT22 | ABC transporter |
|  | K02040 | *pstF* | lp_0733 | F9ULW2 | Phosphate ABC transporter |
|  | K05815 | *ugpE* | lp_1326 | F9UN97 | Glycerol-3-phosphate ABC transporter |
|  | K02012 | *lp_1749* | lp_1749 | F9UPA3 | Iron ABC transporter |
|  | K02011 | *lp_1748* | lp_1748 | F9UPA2 | Iron ABC transporter |
|  | K02037 | *pstD* | lp_0747 | F9ULX4 | Phosphate transport |
|  | K16012 | *cydD* | lp_1129 | F9UMT5 | Cytochrome D ABC transporter |
|  | K16013 | *cydC* | lp_1128 | F9UMT4 | Cytochrome D ABC transporter |
|  | K01990 | *lp_1335* | lp_1335 | F9UNA5 | ABC transporter |
|  | K01992 | *drrB* | lp_1909 | F9UPN2 | Transport permease |
|  | K05816 | *ugpC* | lp_1324 | F9UN95 | Glycerol-3-phosphate ABC transporter |
|  | K06148 | *plnG* | lp_0423 | F9UU09 | Bacteriocin ABC-transporter |
| Fatty acid biosynthesis (12 genes/20 total genes) | K00208 | *fabI* | lp_1681 | F9UP45 | Enoyl-[acyl-carrier-protein] reductase |
|  | K01963 | *accD2* | lp_1679 | F9UP43;Q88WG0 | Acetyl-coenzyme A carboxylase |
|  | K02160 | *accB3* | lp_0362 | F9UTV9 | acetyl-CoA carboxylase |
|  | K01071 | *lp_0708* | lp_0708 | F9ULU3 | Acyl-[acyl-carrier protein] thioesterase |
|  | K00648 | *fabH2* | lp_1671 | F9UP35;Q88WG8 | 3-oxoacyl-[acyl-carrier-protein] synthase |
|  | K01961 | *accC2* | lp_1678 | F9UP42 | Acetyl-CoA carboxylase |
|  | K02372 | *fabZ2* | lp_1677 | F9UP41 | hydroxymyristoyl-[acyl carrier protein] dehydratase |
|  | K02160 | *accB2* | lp_1676 | F9UP40 | acetyl-CoA carboxylase |
|  | K01962 | *accA2* | lp_1680 | F9UP44 | Acetyl-CoA carboxylas |
|  | K09458 | *fabF* | lp_1675 | F9UP39 | 3-oxoacyl-[acyl-carrier-protein] synthase |
|  | K00059 | *fabG1* | lp_1674 | F9UP38 | 3-oxoacyl-[acyl-carrier protein] reductase |
|  | K00645 | *fabD* | lp_1673 | F9UP37 | Malonyl CoA-acyl carrier protein transacylas |

KEGG terms and metabolic pathways were enriched with significantly up-regulated and down-regulated genes (Enrichment with ENSG option with q < 0.04 with Ben. Ho. FDR).

**Supplementary Table S2. Primer sequences used in this study.**

| Gene Name | Sequences | Gene Name | Sequences |
| --- | --- | --- | --- |
| *lp_3278* | 5'-TCTTCGGCGTCCCTTCAAAA-3' | *lp_0018* | 5'-AGTAGCGGCGACGTCATTAG-3' |
|  | 5'-ACCACGAAGCGAATCCAAGT-3' |  | 5'-GCCTGAGCCTTCTGCCAATA-3' |
|  |  |  |  |
| *IrgA* | 5'-CGTGTTAAAGGCAGAAGGCG-3' | *pts4ABC* | 5'-AGCCTTCTGTGGTGTTACCG-3' |
|  | 5'-CGTTCGACGAGCAATGAACC-3' |  | 5'-TCAGTGAACCCGTAAAGCCC-3' |
|  |  |  |  |
| *mntH3* | 5'-ATTCCCGTCCTCATTTGCGT-3' | *lp_3177* | 5'-GTCAGCAATCTTGGCGTTGG-3' |
|  | 5'-ACATGGAGAATGGAAGGGCG-3' |  | 5'-GTCAGCAATCTTGGCGTTGG-3' |
|  |  |  |  |
| *livA* | 5'-GCTTCGGCAACGAACAAAGT-3' | *lp_ 3178* | 5'-AAAATCAAGGCCGCCGGTAA-3' |
|  | 5'-TAGCCGAGCCTTCCTTTTCG-3' |  | 5'-TCTGCTTTGGCTTGACCAGT-3' |
|  |  |  |  |
| *mtsC* | 5'-TCTTGCATCAGTGGCGTGAT-3' | *lp_3050* | 5'-GCTGCTACGGTCGCTATCAA-3' |
|  | 5'-CGTTCAATGTTTGCGGGTGT-3' |  | 5'-CAGAACCGTTTGGCCTTGAC-3' |
|  |  |  |  |
| *dapA2* | 5'-TTCTCGTTTACACGGGCGAA-3' | *lp_3014* | 5'-CGTGAATCCAACTGGCAACC-3' |
|  | 5'-CGCATCGTCGCCATTTCATT-3' |  | 5'-CAACGGCAGCGTTAACTTGT-3' |
|  |  |  |  |
| *guaC* | 5'-CAACTAAGGTTGGCGTTGGC-3' | *lp_2810* | 5'-ACTTAAAGCGACGCGACGTA-3' |
|  | 5'-ACCGTCTGCGATGATTGGTT-3' |  | 5'-CGAGCCACTGGTTTTGTTCG-3' |
|  |  |  |  |
| *purA* | 5'-AACGGTCAACGCGTCCTATT-3' | *lp_2809* | 5'-GCCAATACACAGGACGTTGC-3' |
|  | 5'-CACCGGCAACTGGGTTAGAT-3' |  | 5'-ACTGAGTTTGCCTGTCCGTT-3' |
|  |  |  |  |
| *lp_2993* | 5'-CCCACGTTCAACCGGACTTA-3' | *lp_0304* | 5'-CTGCTGCTCAGACGACTCAA-3' |
|  | 5'-AATCGGTGCGTATTTTGCCA-3' |  | 5'-CAGCGGAGTAGTCACCGTTT-3' |
|  |  |  |  |
| *araT2* | 5'-GGGATTCATCATGGCCCCAA-3' | *lp_0302* | 5'-AGCCAAATGCAATCACGGAC-3' |
|  | 5'-GTTTGACGACGTAATCGCGG-3' |  | 5'-AACGGCAGCGTTAACTTGGT-3' |
|  |  |  |  |
| *cps4E* | 5'-GAACGGCCAGAACTAACCGA-3' | *lp_3421* | 5'-ATTCACGCAAGCTGCCTTTG-3' |
|  | 5'-TCGTACTGACACTTGGCACC-3' |  | 5'-GTATTGGCCACCGCCTAAGT-3' |
|  |  |  |  |
| *aroI* | 5'-GGGCACTGATTGCGTTGAAG-3' | *treA* | 5'-CCGTGGTTGACACCGACTAA-3' |
|  | 5'-AACCACTGCTGAATTTGGCG-3' |  | 5'-GGATCATCTAAGCGCCACGA-3' |
|  |  |  |  |
| *mapB* | 5'-ACCGTATATCAAGCAGGCGG-3' | *cbs* | 5'-GTGGGACATTTGCCGGAGTA-3' |
|  | 5'-CTAGAATGGCATGGACGGCT-3' |  | 5'-ATTCCCTCGGTACGATGTGC-3' |
|  |  |  |  |
| *copB* | 5'-CCATTGGGTCTGGGACTGAC-3' | *cblB* | 5'-CCAGGAATGAACCCCCAGAC-3' |
|  | 5'-AATAAAGCCGAGTGGTGCGA-3' |  | 5'-CTGTAGCCGAATTGTGCGTG-3' |
|  |  |  |  |
| *copA* | 5'-TCACCCACAAGTGACCGATG-3' | *cysE* | 5'-GTTTGTCGCGGTGCTTTCAT-3' |
|  | 5'-CCCTCGATGGCTTGGAAGTT-3' |  | 5'-GCGGGAACGCTGTCTAAAAC-3' |
|  |  |  |  |
| *lp_0783* | 5'-TTCAACGACCCAATCTCGCA-3' | *16S* | 5'-AGC AGT AGG GAA TCT TCC A-3' |
|  | 5'-TCCTGGTTTTGGGCCTTGTT-3' |  | 5'-CAC CGC TAC ACA TGG AG-3' |
|  |  |  |  |
| *pnuC1* | 5'-TCGGCGACACTAGCAATGTT-3' |  |  |
|  | 5'-TGCTTGTTCTTCGGCCATCA-3' |  |  |

Primers were designed based on nucleotide sequences from *L. plantarum* of NCBI database (GCF_000203855.3, *L. plantarum* WCFS1).
